# Immunity to Sexually Transmitted Bacterial Infections of the Female Genital Tract: Toward Effective Vaccines

**DOI:** 10.3390/vaccines12080863

**Published:** 2024-08-01

**Authors:** Kacy S. Yount, Toni Darville

**Affiliations:** Department of Pediatrics, The University of North Carolina at Chapel Hill, Chapel Hill, NC 27599, USA; kyount@email.unc.edu

**Keywords:** sexually transmitted infections (STIs), *Chlamydia trachomatis*, *Neisseria gonorrhoeae*, *Treponema pallidum*, immunology, T cells, B cells, vaccines, bacterial pathogens, reproductive health

## Abstract

Sexually transmitted infections (STIs) caused by bacterial pathogens *Chlamydia trachomatis*, *Neisseria gonorrhoeae*, and *Treponema pallidum* present significant public health challenges. These infections profoundly impact reproductive health, leading to pelvic inflammatory disease, infertility, and increased susceptibility to other infections. Prevention measures, including antibiotic treatments, are limited by the often-asymptomatic nature of these infections, the need for repetitive and continual screening of sexually active persons, antibiotic resistance for gonorrhea, and shortages of penicillin for syphilis. While vaccines exist for viral STIs like human papillomavirus (HPV) and hepatitis B virus (HBV), there are no vaccines available for bacterial STIs. This review examines the immune responses in the female genital tract to these bacterial pathogens and the implications for developing effective vaccines against bacterial STIs.

## 1. Introduction

Sexually transmitted infections (STIs) are a significant public health burden, both in the United States and worldwide [1]. Direct impacts of STIs on sexual and reproductive health include stigmatization, pelvic inflammatory disease (PID), pregnancy complications, infertility, and increased susceptibility to cervical cancer and HIV. In the United States in 2018, there were an estimated 68 million STIs and 26 million new infections. Bacterial STIs, including chlamydia, gonorrhea, and syphilis, accounted for 4 million, 1.6 million, and 146,000 infections, respectively, and contributed to over USD 1 billion in direct medical costs [2]. Nearly half of these STIs occurred in young people (ages 15–24) and care for women with STIs represented nearly 75% of all non-HIV STI-related medical costs.

Increased prevention measures are important to combat the STI epidemic. Bacterial STIs are curable with antibiotics. However, shortages of penicillin amid rising syphilis rates and antibiotic resistance to gonorrheal infections are increasing problems [3]. Further, most bacterial STIs are asymptomatic, limiting the number of people who seek antibiotic treatment and increasing transmission of the pathogens [2]. Current preventive measures include condom use and increased diagnostic surveillance. While vaccines have been developed against the viral STIs human papillomavirus (HPV) and hepatitis B virus (HBV), there are no vaccines against bacterial STI pathogens. Designing and implementing effective vaccines against bacterial STIs is a major research effort and depends heavily on our understanding of the immune response to these pathogens in the female genital tract. In this review, we will discuss the pathogens that cause bacterial STIs, adaptive and innate immunity to these pathogens, current vaccine strategies, and existing gaps in knowledge and implications for vaccine design (Table 1).

## 2. Bacterial STIs of the Female Genital Tract

### 2.1. Chlamydia

*Chlamydia trachomatis* is a Gram-negative obligate intracellular bacterium and the causative agent of chlamydial infections. Serovars A–C cause conjunctival infections of the eye, D–K cause the most common urogenital tract infections in the US, and L1–L3 cause lymphogranuloma venereum (LGV), a mucosally invasive infection [42]. *C. trachomatis* has a unique biphasic developmental cycle in which infectious elementary bodies [EBs) invade host epithelial cells, form an inclusion in which they transition to reticulate bodies for replication, and transition back to EBs for cell lysis or extrusion for cell attachment and re-initiation of the cycle [4]. *C. trachomatis* urogenital infections are detected in almost twice the number of women as men in the US [43], likely due to increased screening in women [44]. Up to 70% of *C. trachomatis* genital tract infections in women, and up to 50% in men, are asymptomatic. Symptomatic infections present with vaginal and/or urethral discharge, painful urination, dyspareunia, lower abdominal or pelvic pain in women, and testicular pain associated with epididymitis in men. Due its more invasive nature, LGV can induce severe proctitis from rectal infection, rectal strictures, and painful lymphadenopathy from lymph node invasion. Infections can also spread from infected mothers to newborns at the time of delivery, causing neonatal conjunctivitis and pneumonia. Antibiotic treatment with doxycycline or azithromycin is effective and antibiotic resistance is rare [45]. However, many asymptomatic infections are untreated and can last up to 4 years [46], resulting in continued asymptomatic transmission contributing to high prevalence of infections. Despite the high rate of asymptomatic infection, up to 40% of untreated infections in women ascend to involve the upper genital tract, causing damaging immunopathology which may lead to PID and reproductive sequelae including chronic pelvic pain, pregnancy complications, and infertility [42].

### 2.2. Gonorrhea

*Neisseria gonorrhoeae* is a Gram-negative diplococcus bacterium and the causative agent of urogenital gonorrheal infections. The organism infects epithelial cells and has virulence factors that allow it to survive inside infiltrating neutrophils via resistance to antimicrobial components in neutrophil granules [47]. Its genome, and the outer membrane proteins it encodes, is highly variable, allowing for immune evasion and complicating diagnostics, antibiotic treatment, and vaccine development [48,49]. Gonorrheal infections in the US are most prevalent among men and most infections are asymptomatic [43]. Urogenital symptoms are similar to those reported above for chlamydial infections. Approximately 10% of women with gonorrhea infections develop PID from infections that ascend to the upper genital tract with potential for consequent reproductive morbidities, as described with chlamydial infection [50]. Unlike chlamydial infections, which remain restricted to the mucosa, gonorrheal infections can disseminate through the bloodstream and result in bacterial sepsis with skin rash and multifocal arthritis, septic arthritis of single joints, endocarditis, and rarely, meningitis and hepatitis. Gonococcal infection can also be transmitted to newborns during delivery from an infected mother and can lead to severe neonatal ocular infection or disseminated infection as seen in adults. Ceftriaxone is the recommended antibiotic treatment and is highly effective [45]. However, antibiotic resistance is a growing concern for gonorrheal infections, as ~50% of isolates from infections in the US in 2022 were estimated to be resistant to at least one antibiotic [43], emphasizing the need for preventive vaccine development.

### 2.3. Syphilis

*Treponema pallidum* is a spirochete and is the causative agent of syphilis infections. The organism infects the epithelium and replicates inside tissues, inducing damaging inflammation [51]. Primary syphilis typically presents as a solitary, painless chancre at the site of infection, usually the genitals, anus, or mouth [52,53]. The chancre appears approximately 3 weeks after exposure and heals spontaneously within 3–6 weeks if untreated. Secondary syphilis occurs weeks to months after the initial infection and is characterized by systemic dissemination of the bacterium. Common clinical manifestations include a diffuse rash that often involves the palms and soles, skin lesions, and swollen lymph nodes. Other symptoms can include fever, fatigue, sore throat, and hair loss. Syphilis infections are sexually transmissible at this stage. Upon resolution, symptoms typically subside during latent syphilis and infections can only be detected during this stage by serologic testing. Tertiary syphilis can develop years to decades after the initial infection. It is characterized by large, destructive lesions affecting the skin, bones, and other tissues, heart problems, and neurosyphilis. Neurosyphilis can present at any stage but is more common in tertiary syphilis, manifesting as muscle weakness, coordination issues, seizures, deafness, and blindness [52,53]. Syphilis can also be passed from mother to child, causing congenital infections which are associated with fetal demise, prematurity, low birth weight, pneumonia, rash, neurologic disease, and bone abnormalities [54,55].

Of the three bacterial STIs discussed in this review, syphilis is the least prevalent in the US. In 2022, the Centers for Disease Control reported 207,255 cases of syphilis, 59,016 (28%) of which were primary and secondary syphilis cases [43]. Cases of syphilis have increased rapidly in recent years, with an increase of 17.3% from 2021 to 2022 [43]. A proportional increase in cases of congenital syphilis infections has also been observed [43,54], emphasizing the need for improved treatment and prevention measures. Penicillin is effective against syphilis infections [45], but asymptomatic untreated infections are common, and the organism can remain latent for long periods of time, increasing the risk of transmission.

## 3. B Cells and Antibody-Mediated Immunity

The primary role of B cells in the immune response against STIs and other pathogens is to secrete antibodies. Naïve B cells encounter antigens that are recognized by the B cell surface receptor. With stimulation by follicular dendritic cells and help from T cells, B cells form germinal centers in draining lymph nodes and undergo affinity maturation and class switching. B cells replicate and develop into short-lived immunoglobulin-secreting plasmablasts, memory B cells, or long-lived plasma cells. Memory B cells are long-lived antigen-specific B cells that are poised to recall and respond upon antigen re-exposure. Long-lived plasma cells home to the bone marrow and secrete antibodies that can last in circulation for years to a lifetime [56].

The five classes of antibody (IgA, IgD, IgE, IgG, and IgM) differ in their effector functions, anatomical distribution, and protection against different pathogens. IgG is the most abundant antibody class in the bloodstream and is involved in systemic immunity via neutralization, opsonophagocytosis, and antibody-dependent cellular cytotoxicity (ADCC).

### 3.1. IgG and Chlamydia Infection

#### 3.1.1. Neutralization

Neutralization is the mechanism by which an antibody recognizes and binds to surface antigens on the pathogen, thereby blocking the ability of the pathogen or a secreted toxin to attach and interact with host cells. Although antibody-mediated neutralization of *C. trachomatis* has been demonstrated in vitro [57], this organism enters the host cell by a multitude of mechanisms including non-receptor–ligand-mediated pinocytosis [58]. There is also a panoply of bacterial surface ligand–host cell receptor-mediated interactions that enhance chlamydial invasion of host epithelial cells [59], serving as an effective evasion mechanism to thwart antibody neutralization. Female mouse models of chlamydial genital infection have revealed that IgG antibodies specific for the chlamydial major outer membrane protein (MOMP) that led to neutralization in vitro lowered the burden but failed to protect against *C. trachomatis* genital tract infection in mice [60,61].

#### 3.1.2. Opsonophagocytosis

Opsonophagocytosis is the coating of a pathogen with antibodies that allows phagocytic cell FcRs to target the pathogen for destruction. Mouse model studies have revealed that chlamydia-specific antibody interactions with phagocytic cells can limit infectious burden when interferon gamma (IFNγ) is present [62,63,64,65]. Antibodies opsonize extracellular chlamydial elementary bodies by binding to a specific antigen epitope via Fab domains. The free Fc domain binds to immune effector cells, leading to opsonophagocytosis, and enhanced bacterial killing.

IFNγ upregulates phagocytic cell FcR expression and is required to enhance chlamydial opsonophagocytosis [66]. Naglak et al. reported that depletion of neutrophils and CD4^+^ T cells led to a chronic infection of significantly greater burden than in mice depleted of CD4^+^ T cells alone [67]. However, *C. trachomatis* can resist neutrophil opsonophagocytosis and killing by chlamydial protease-like activity factor (CPAF), which suppresses oxidative bursts, interferes with chemical-mediated activation, and prevents neutrophil extracellular trap (NET) formation [5]. Using B-cell deficient mice that had resolved a primary infection and were subsequently depleted of CD4^+^ T cells, they showed that transfer of Fab fragments from immune mice failed to provide any protection against chlamydial challenge infection, demonstrating that antibody-mediated protection is dependent on IgG with intact Fc [67].

#### 3.1.3. Antibody-Dependent Cellular Cytotoxicity (ADCC)

Antibody-dependent cellular cytotoxicity (ADCC) is a third mechanism whereby antibodies mediate pathogen killing. It typically involves natural killer (NK) cell recognition of antibody-opsonized cells expressing non-self-antigens, but can also involve other myeloid cells. NK cells bind the Fc portions of antibodies and trigger the release of cytotoxic molecules, killing the infected target cell. Although NK cell depletion prolongs primary murine chlamydial infection, it leads to a shift towards a Th2 response [68], and depletion of NK cells during secondary infection has no effect on time to resolution in the B cell-deficient, CD4-depleted challenge model [67]. However, combined depletion of NK cells and CD4 T cells led to enhanced burden over CD4 depletion alone [67].

In vitro studies show that anti-chlamydial antibodies can increase both macrophage killing of infected epithelial cells and macrophage inhibition of productive growth of chlamydiae in co-cultures [69]. Anti-chlamydial antibodies can also boost antigen presentation, thereby augmenting protective memory T cell responses active during repeat infections [69]. Despite these protective mechanisms, B cells are exceedingly rare in the female genital tract, making up less than 1% of leukocytes in healthy cervical tissue compared to T cells that make up ~35% of leukocytes [70]. Some B cells in the cervix have been shown to form follicle-like structures that are closely associated with aggregates of T cells, while other CD38+ immunoglobulin-producing B cells are scattered among the lamina propria of the cervix and vagina [71]. IgG antibody produced by circulating B cells that transudates into genital secretions is a primary source of B cell-derived protection against pathogens of the female genital tract.

#### 3.1.4. FcRn Transcytosis

IgG can be transported from the apical to the basolateral surface of mucosal epithelial cells and into the tissue through transcytosis. The neonatal Fc receptor (FcRn) is found throughout the mucosal tissues of mammals, including on epithelial, endothelial, and antigen-presenting cells [72]. FcRn binds to IgG antibodies under acidic conditions (pH 5–6.5) and releases them at neutral pH (pH 7.4). This allows FcRn to transport IgG antibodies both into and out of the lumen of the male and female reproductive tracts, depending on the pH [73,74].

Whether or not transcytosing antibodies exacerbate or inhibit chlamydia infection in vitro is dependent upon antibody specificity [75]. IgG specifically targeting chlamydial MOMP increased FcRn-dependent uptake and translocation of chlamydial elementary bodies in polarized male epididymal epithelia. In contrast, FcRn-internalized IgG, which targets a chlamydial inclusion membrane protein, IncA, led to reduced in vitro infection. Further, it has been shown that additional inclusion membrane proteins that are cytoplasmic facing can be bound by intracellular IgG, and microinjection of an anti-Inc (CT229) antibody into infected cells inhibited chlamydial infection [76,77].

### 3.2. IgA and Chlamydia Infection

Secretory IgA (SIgA) is found predominantly in mucosal secretions and functions to neutralize pathogens by blocking their attachment to mucosal epithelial cells and by preventing their entry into tissues. In human cervical mucus, IgG predominates over IgA in abundance, which directly contrasts with other typical external secretions, such as saliva, tears, milk, and intestinal fluids, in which SIgA is the dominant isotype [78,79]. Although IgA is known to protect against a variety of mucosal pathogens [80], IgA-deficient mice resolve primary and secondary chlamydial genital infections similarly to IgA-sufficient mice [81]. It is possible that any contribution from IgA is masked by redundancy and high levels of IgG transcytosed via FcRn.

### 3.3. Chlamydia Antibody in Humans

Human data support a role for antibodies in limiting chlamydial burden but not reinfection. A majority of *C. trachomatis*-infected women and at least 50% of men develop detectable levels of anti-*C. trachomatis* serum IgG and IgA [82]. Additionally, *C. trachomatis*-specific IgG and IgA antibodies were more frequently found in female genital tract secretions when compared to serum [83]. Despite this, rates of repeat infections are high [84,85,86,87], failing to support a role for *C. trachomatis*-specific antibodies in resistance to reinfection.

We analyzed the relationship of serum anti-*C. trachomatis* IgG determined by microimmunofluorescence (MIF) assay to whole EBs in a cohort of 225 young women who were highly sexually exposed to *C. trachomatis,* with 83% being seropositive at enrollment [6]. Although we found an inverse relationship of antibody titers with cervical chlamydial burden as determined by quantitative PCR, there was no relationship determined between antibody titer and detection of endometrial chlamydial infection, indicating that antibodies failed to limit risk for upper genital tract infection and potential for reproductive sequelae. We also determined that both serum and cervical anti-*C. trachomatis* IgG correlated with increased risk of incident infection, with hazard ratios increasing 3.6-fold and 22.6-fold with each unit of serum and cervical IgG, respectively [6]. We went further and used a whole-proteome *C. trachomatis* array to screen serum samples for IgG to specific chlamydial proteins but were unable to identify protein-specific antibody responses that correlated with reduced risk of reinfection [7]. In contrast, in this same cohort, we determined that increased frequencies of chlamydial antigen-specific IFNγ-producing CD4 T cells were associated with reduced risk of reinfection [88]. Women with the highest anti-chlamydial antibody responses may be Th2-skewed, resulting in inadequate IFNγ and cell-mediated responses that are key for protection. Indeed, several studies correlate high titers of serum anti-chlamydial IgG with complications of infection [89,90,91]. As opposed to other mucosal pathogens, where antibodies in mucosal secretions function as a first line of defense [92], data from human studies of chlamydial genital tract infection fail to support a role for antibodies in the limitation of spread of infection to the upper genital tract in persons with a uterus, or acquisition of infection regardless of gender.

### 3.4. Antibody in Gonorrhea Infection

*N. gonorrhoeae* has a remarkable ability to modify its surface antigens, which differ not only between strains but also within the same strain over time. The organism also practices phase variation, which allows it to switch between phenotypic states rapidly and reversibly, altering expression of surface proteins over time [18]. Both mechanisms make it extremely difficult for antibody responses elicited by a primary gonorrhea infection to protect against a secondary infection [32]. These aspects, and the consideration that multiple other species of *Neisseria* also possess gonococcal antigens, further complicate serological studies in humans. Even so, both antibody and cell-mediated cytokine responses to documented, uncomplicated gonococcal infection are weak and wane after only a few weeks in both women and men [93,94]. Low levels of gonococcal antibody have been partially explained by the manipulation of the immune response by *N. gonorrhoeae.* One way *N. gonorrhoeae* suppresses antibody responses is by inducing IL-10-producing type 1 regulatory T cells. In a mouse model, these cells contributed significantly to the suppression of adaptive immunity, whereas their absence allowed antibody responses to develop [19]. *N. gonorrhoeae* encodes an IgA1 protease that masks epitopes by cleaving antigen-bound IgA [22]. It also has multiple mechanisms to evade complement, including sialylation of lipooligosaccharides (LOS) to prevent recognition by complement components [20] and binding of factor H and C4b-binding protein (C4BP) via surface proteins such as factor H binding protein (fHbp) and PorB to inhibit complement activation [21]. Between surface antigen variation and humoral immune evasion, it is not surprising that infections fail to elicit protective immunity upon re-exposure.

### 3.5. Antibody in Syphilis Infection

IgG and IgM against *T. pallidum* proteins have been detected in experimentally infected rabbits [95,96] and in humans with primary, secondary, and late-stage syphilis [97]. Partial immunity to *T. pallidum* challenge was conferred by adoptive transfer of immune serum in experimental infection of rabbits and hamsters [98,99], with the main mechanism of protection being antibody opsonization and induction of phagocytosis and killing by macrophages [35,36,37]. However, T. pallidum isolates from humans had varied capacities for binding opsonic antibodies, suggesting a potential evasion mechanism [100]. The high rates of syphilis reinfection despite detection of *T. pallidum* specific antibodies in previously infected persons indicates ineffective antibody-mediated protection is induced after natural infections. Specifically, antigenic variation of *T. pallidum* surface protein TprK can evade antibody-mediated immunity [34].

## 4. T Cell-Mediated Immunity

T cells encounter peptide antigens in the context of major histocompatibility complex (MHC) molecules on the surface of antigen-presenting cells such as dendritic cells, macrophages, and B cells. Activated T cells proliferate and differentiate into effector T cells which contribute to pathogen elimination. The main role of CD4^+^ T-helper cells is to secrete cytokines that are crucial for activating and regulating both innate and adaptive immune responses [101]. T-helper 1 (Th1) cells secrete IFNγ, TNFα, TNF, and IL-2, and contribute to elimination of viruses and intracellular bacteria by activating macrophage phagocytosis and enhancing CD8^+^ T cell cytotoxicity. Th2 cells secrete IL-4, IL-5, IL-13, and IL-10 and contribute to defense against extracellular pathogens and secreted toxins by stimulating B cells to produce antibodies and recruiting and activating eosinophils and mast cells. Th17 cells are highly plastic and secrete IL-17, IL-21, and IL-22 to promote inflammation and recruitment of neutrophils to the site of infection. Th17 cells give rise to long-lived resident memory T cells that are essential for protection against various mucosal pathogens [102], including STIs, but can also be pathogenic. T follicular helper (Tfh) cells secrete cytokines such as IL-21 and IL-4 and are specialized in providing help to B cells in the germinal centers of secondary lymphoid organs, in turn promoting antibody class switching, affinity maturation, and the generation of long-lived plasma cells and memory B cells [101]. Regulatory T (Treg) cells produce IL-10 and TGF, functioning as important negative regulators of the immune response to prevent over-activation and unchecked inflammation. CD8^+^ cytotoxic T lymphocytes (CTL) recognize and directly kill infected cells displaying foreign antigens by releasing cytotoxic granules containing perforin and granzymes or by inducing apoptosis in target cells through the Fas-Fas ligand pathway.

### 4.1. T Cells and Chlamydia Infection

**Figure 1:**
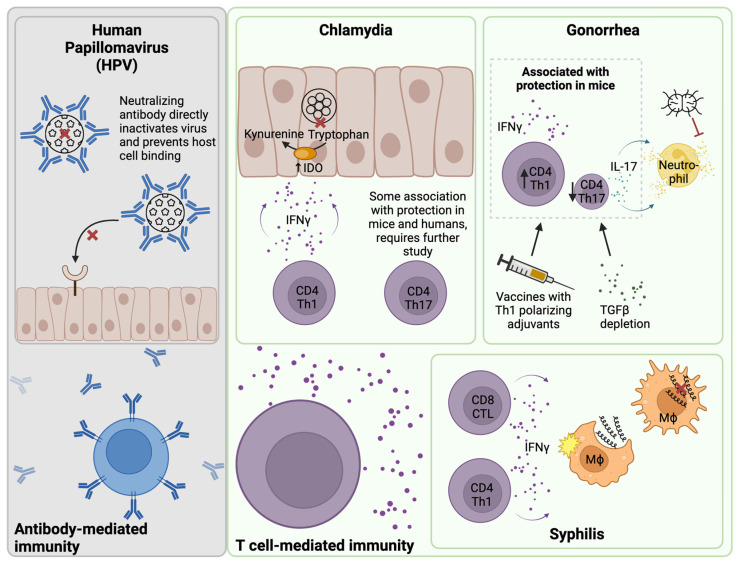
T cell-mediated immunity for bacterial STIs of the female genital tract. Immunity to human papillomavirus (HPV) is mediated by a neutralizing antibody, which directly inactivates the virus and prevents the virus from binding to and entering host epithelial cells. In contrast, evidence points toward T cell-mediated immunity for bacterial STIs of the female genital tract including chlamydia, gonorrhea, and syphilis. In the context of chlamydia infection, CD4 Th1 cells are an abundant source of IFNγ. IFNγ induces epithelial cells to upregulate indoleamine 2,3-dioxygenase (IDO), which starves chlamydia of the essential nutrient tryptophan by metabolizing it to kynurenine. CD4 Th17 cells are also associated with protection in mice and humans, but further study is required. Gonorrhea induces a strong Th17 response, which supports neutrophil influx. This Th17 response is favorable for gonorrhea survival because the organism can resist neutrophil killing both intracellularly and extracellularly. Immunization with vaccines containing Th1-polarizing adjuvants or depletion of TGFβ in mice results in suppression of Th17 responses and induction of Th1 responses, which are associated with greater protection. In the context of syphilis infection, IFNγ from T cells and innate cells supports recruitment and activation of macrophages, which phagocytose and mediate killing of the organism. Created with Biorender.

Studies have shown that SCID, RAG-1^−/−^, TCRα^−/−^, CD4^−/−^, and MHCII^−/−^ mice display delayed or failed resolution of chlamydial primary infections, while MHCI-deficient (b2M^−/−^), B cell-deficient (μMT), and CD8^−/−^ mice resolve infection with comparable kinetics to wild-type, demonstrating that CD4^+^ T cells are key mediators of chlamydial host defense [62,103,104,105,106]. Gene-knockout, antibody depletion, and adoptive transfer studies in mice indicate Th1 cells and their defining production of IFNγ are key mediators for resolving primary infections [10,107,108]. However, this mechanism may be independent of expression of T-bet, the transcription factor that putatively defines Th1 cells [109]. CD8-derived IFNγ can also contribute to resolution of *C. trachomatis* infection in mice [110]. In humans, IFNγ mediates *C. trachomatis* killing by inducing indoleamine-2,3-dioxygenase (IDO) production in epithelial cells, which catabolizes tryptophan, thereby starving *C. trachomatis* of this essential nutrient (Figure 1) [9]. In mice epithelial cells, however, IFNγ induces expression of p47 GTPases, but not IDO, demonstrating host infection tropism and defining an important difference to consider when utilizing mouse models of chlamydia infection [111].

Adoptive transfer of a chlamydia-specific Th2 clone did not protect against murine genital challenge and T cells displayed reduced trafficking to the genital mucosa [112]. Th17 cells can be protective or pathogenic in contexts of infection and disease [113,114]. One study found that blocking IFNγ signaling promoted tissue damage by greatly enhancing chlamydia-specific Th17 responses [115]. Another study found that IL-17 supported neutrophil influx, a major contributor to chlamydial pathology, but also protective Th1 responses during murine chlamydial infection [116]. IL-17 was not required for infection resolution [116]. We detected both chlamydia-specific IFNγ and IL-17-producing CD4^+^ T cells in *C. trachomatis*-exposed adolescents and adults [88,117]. Increased frequencies of Th1 and Th17 cells were also found to be associated with reduced reinfection in a highly exposed cohort of *C. trachomatis*-infected women, while increased frequencies of Th2 cells were found to be associated with increased reinfection over time [8]. Together, these results suggest that Th1 responses drive protective immunity to *C. trachomatis*, while Th2 responses are not protective, and the role of Th17 responses requires further study (Figure 1).

### 4.2. T Cells and Gonorrhea Infection

Studies in mice have shown that *N. gonorrhoeae* genital tract infection drives a Th17 cytokine profile and suppresses Th1 or Th2 responses through the induction of TGFβ [23]. This immune evasion strategy results in recruitment of neutrophils, which *N. gonorrhoeae* resists both intracellularly and extracellularly [24]. Indeed, TGFβ depletion in mice resulted in suppressed Th17 responses and enhanced Th1 and Th2 responses to *N. gonorrhoeae* infection, which were associated with increased protection against subsequent challenge (Figure 1) [25]. Two of the characteristic cytokines produced by Th17 cells are IL-17 and IL-22. Interestingly, IL-17 depletion resulted in prolonged gonorrheal infection in mice [118] while IL-22 depletion resulted in accelerated clearance [119], demonstrating divergent roles for these two Th17 effectors in the context of gonorrheal immunity. Studies in gonococcal-infected humans found increased IL-17, IL-23, and IFNγ in serum [120], which suggests that Th17 responses are also activated in humans. In summary, strong evidence exists for Th1 cells to play a role in protection for both chlamydial and gonococcal infections, and recent data suggest Th17-lineage cells may contribute to resistance to chlamydial reinfection and may be involved in resolution of gonococcal infection.

### 4.3. T Cells and Syphilis Infection

Experimental infection of male rabbits with *T. pallidum* subsp. *pallidum* elicited a CD4^+^ Th1-dominant response in testicular lesions [121,122]. Detection of high levels of IL-2, IFNγ, and IL-12 alongside low levels of IL-4, IL-5, and IL-13 transcripts by RT-PCR in primary and secondary human lesion biopsies also support a Th1 profile [123]. CD8^+^ T cells have also been detected in syphilis lesions in humans [124]. Salazar et al. reported that CD4 and CD8 T cells from blister fluid of males and females with secondary syphilis expressed higher levels of CCR5, a mucosal homing marker, and CD45RO, a marker of T cell memory, than T cells in peripheral blood [125]. The relative contributions of T cell subsets to protection against syphilis infection is unclear, although it is generally thought that production of IFNγ by innate cells and CD4 or CD8 T cells is important for recruiting and activating macrophages, which facilitate *T. pallidum* killing (Figure 1) [35,41]. Further, many of the rabbit model studies of T cell responses were limited to evaluation of male testicular lesions, and studies in the context of the female genital tract are very limited.

### 4.4. Resident Memory T Cells in the Female Genital Tract

Most activated T cells undergo apoptosis after resolution of a primary immune response to a specific pathogen, but some differentiate into memory cells, which persist in the periphery and quickly respond to subsequent encounters with the same pathogen. Central memory T (TCM) cells primarily reside in secondary lymphoid organs, where they serve as a long-term reservoir of antigen-specific T cells, remaining poised to proliferate and differentiate into effector cells capable of trafficking to the site of infection upon reactivation [126]. They typically express high levels of the lymph node-homing receptor CD62L (L-selectin) and the chemokine receptor CCR7, which facilitate their migration and retention within secondary lymphoid organs. Effector memory T (TEM) cells lack CD62L and CCR7 expression, circulating between peripheral blood and mucosal tissues, where they are primed to respond immediately to reactivation.

Resident memory T (TRM) cells are a subset of TEM that do not circulate but are retained in mucosal tissues and barrier sites. TRM cells contribute to frontline defense in both mice and humans against a variety of mucosal pathogens [127,128]. TRM cells are classically defined by expression of CD69, which inhibits the sphingosine-1 phosphate receptor (S1PR), a molecule required for egress from tissues or secondary lymphoid organs to the circulation [129,130,131].

Studies have documented TRM cells in the genital tracts of healthy women [132,133]. CD8 T cells predominated in the vagina while CD4 T cells predominated in the ectocervix and endocervix [132]. CD4 and CD8 T cells in the genital tract were primarily effector memory T cells as evidenced by lack of CD45RA and CCR7 [132,133]. CD4 and CD8 TRM cells in the female genital tract expressed higher levels of the following markers than CD4 and CD8 T cells in peripheral blood: CD69 and CD103, putative markers of TRM cells; PD-1, a marker upregulated on T effector cells in peripheral and mucosal tissues [134]; and CCR5, an important factor for healthy barrier immunity in the genital tract [135,136]. CD4 TRM cells expressed lower levels of CD103 than their CD8 counterparts [133], but CD69^+^CD103^+^ CD4 TRM cells were still detected across the vagina, ectocervix, and endocervix [132], with higher frequencies detected in the vagina. However, most CD4 TRM cells in all three tissues were CD69^+^CD103^−^ [132]. Of note, CD69^−^CD103^−^ T cells from genital tract tissues were transcriptionally distinct from CD69^−^CD103^−^ T cells from peripheral blood, by demonstrating enrichment of cell migration and localization pathways [133]. A recent study showed that TRM cells are not only distinct between blood and mucosal sites, but are also distinct between different mucosal tissues in the same individuals including lung, skin, and jejunum [137]. Pattacini et al. aligned gene expression of cervicovaginal TRM cells with a previously published dataset comparing gene signatures of human spleen and lung tissues [134], and found both gene expression signatures that were common and unique to cervicovaginal TRM cells [133]. These findings support the further study of TRM cells in the female genital tract.

This review focuses on immunity to bacterial STIs, but much of the foundational work regarding TRM cells in the context of STIs of the female genital tract comes from studies of herpes simplex virus (HSV) in mice and humans [138,139,140,141,142,143,144,145,146]. Work in the contexts of human papillomavirus (HPV) and cervical cancer [147,148,149], lymphocytic choriomeningitis virus (LCMV) [147,148,149], and human immunodeficiency virus (HIV) [150,151,152,153,154,155,156,157] has also demonstrated the importance of TRM cells in the female genital tract in the context of viral infections. While CD8^+^ TRM cells are key antiviral effectors [158], CD4^+^ TRM cells are likely the main contributors to defense against bacterial STIs such as chlamydia.

Before the term TRM cells was coined, clusters of T cells were reported in genital tract tissues of chlamydia-infected convalescent mice [159], in chlamydia-infected macaques [160], and in cervical and endometrial samples from women with *C. trachomatis* infections [161,162]. Early studies also demonstrated the importance of IFNγ and T cell contact with epithelial cells for nitric oxide-mediated killing of *Chlamydia* in the female genital tract of mice [163,164]. Additionally, T cells migrating to the female mouse genital tract following chlamydial infection were mostly CD4^+^ and expressed increased mucosal homing molecules LFA-1 (CD11a) and α4β7 [165,166].

Studies in more recent years have investigated the role of chlamydia-specific TRM cells elicited by vaccination in mice. Stary et al. showed that intranasal or intrauterine but not subcutaneous immunization of female mice with UV-inactivated *C. trachomatis* (UV-Ct) complexed with charge-switching synthetic adjuvant particles (cSAP) induced CD4 TRM cells in the genital tract, with increased expression of IFNγ, TNFα, and IL-2 that rapidly expanded upon challenge, resulting in accelerated bacterial clearance and reduced oviduct pathology [108]. Two “waves” of protective T cells were induced, including TRM cells retained in the genital tract after mucosal immunization and circulating effector cells that were quickly recruited to the genital tract following challenge. The circulating memory subset contributed to the overall recall response, but its protective role was suboptimal in the absence of TRM cells, as evidenced by parabiotic mouse experiments. Importantly, mucosal immunization of mice with unadjuvanted UV-Ct exacerbated infection via induction of FoxP3^+^ CD4 regulatory T cells, demonstrating that vaccine platforms and adjuvants are important considerations for effective vaccine design. Nguyen et al. detected IFNγ- and IL-17-producing TRM cells in female genital tracts of *C. trachomatis*-challenged mice that had been immunized subcutaneously with CAF01-adjuvanted variable domain 4 (VD4) of the major outer membrane protein (MOMP) [167]. The same group found that simultaneous subcutaneous and intrauterine immunization with the same vaccine was required to elicit CD4 Th1 and Th17 TRM pre-challenge [168]. Notably, nearly 40% of Th17 TRM co-expressed IL-17 and IFNγ by 14 days post-infection, suggesting that Th17 cells may contribute to IFNγ-mediated protection against chlamydial infection. Overall, it has been shown that mucosal vaccines elicit CD4 TRM cells in the genital tracts of mice and that these cells likely contribute to accelerated resolution of chlamydial infection upon challenge. Further research is needed to elucidate genital tract TRM cells’ responses after natural chlamydial infection, both in mouse models and in human cohorts.

Notably, studies of resident memory T cells in the context of gonorrhea or syphilis infection have not been reported in either mice or humans but will be important for understanding their contributions to infection resolution with these pathogens.

## 5. Innate Lymphocyte-Mediated Immunity

Although not a part of the trained adaptive response, the role of innate lymphocytes in protection against bacterial STIs is worth mentioning.

### 5.1. NK Cells

Upon pathogen recognition, natural killer (NK) cells release cytotoxic granules containing perforin and granzymes, inducing apoptosis in the target cell. They also secrete IFNγ and TNFα, which are important effectors and regulators of downstream immune responses. An early study reported that NK cells were recruited to the female genital tracts of mice as early as 12 h post-infection with *C. muridarum,* and that depletion of NK cells resulted in reduced IFNγ and a Th2-skewed immune response [68]. Later studies corroborated that depletion of NK cells exacerbated *C. muridarum* infection in mice and further demonstrated that IFNγ from NK cells promotes IL-12 production by dendritic cells, which then supports a strong Th1 response [169]. Depletion of NK cells also impaired production of IFNγ by memory T cells in respiratory *C. muridarum* infection [170]. These studies suggest that the early presence of NK cells and their production of IFNγ could both directly inhibit chlamydial growth by tryptophan depletion [9] and support Th1 responses associated with chlamydial protection [107].

### 5.2. Helper Innate Lymphoid Cells (ILCs)

Helper innate lymphoid cells (ILCs) recognize pathogens through Toll-like receptors (TLRs) and pattern recognition receptors (PRRs) and secrete effector cytokines that modulate the downstream immune response like T-helper cells. They are present in lymphoid and non-lymphoid tissues, but are most abundant in mucosal tissues, where they play an important role in pathogen defense against lung and gut pathogens [171]. ILCs are present in the female genital tracts of healthy humans and mice and are dominated by the ILC3 subtype [172,173]. ILC3s produce IL-17 and IL-22 and express transcription factors including RORγt, RORα, and HIF1, which inhibit their plasticity toward ILC1s, which express T-bet, in conjunction with Aiolos and Bcl6 [174]. The contribution of ILCs to protection against STIs of the female genital tract is largely unknown. Initial studies have found that ILCs accumulate in the oviducts of mice infected with *C. muridarum* [175] and that transfer of ILC3-like cells to the mouse colon prevents gastrointestinal colonization by *C. muridarum* [176]. The contribution of ILCs in the context of gonorrhea or syphilis infection is unknown. Significant further study will be required to elucidate the protective capacity of ILCs in the context of bacterial STIs of the female reproductive tract.

### 5.3. Mucosal-Associated Invariant T (MAIT) Cells

Mucosal-associated invariant T (MAIT) cells are a population of unconventional innate-like T cells that have unusual specificity for microbial riboflavin-derivative antigens presented by the MHCI-like protein MR1 [177]. They are defined by surface expression of the semi-invariant T cell receptor Vα7.2 and high expression of C-type lectin CD161. They can be activated by TCR-dependent and TCR-independent mechanisms, secrete cytokines including IFNγ, TNFα, IL-17, and IL-22, and are involved in defense against a variety of mucosal pathogens [178]. Gibbs et al. were the first to report MAIT cells in the human female genital tract [179]. MAIT cells were present in the endometrium, endocervix, transformation zone, and ectocervix, as were MR-1^+^ antigen-presenting cells. MAIT cells accounted for ~1.0% of T cells in the endometrium, ~1.8% of T cells in the cervix, and ~2.1% of T cells in the blood. CD8^+^CD4^−^ MAIT cells predominated over CD4^+^ and CD8^−^CD4^−^ MAIT cells in all three tissues. MAIT cells isolated from the genital tract produced IL-17- and IL-22-biased responses upon ex vivo stimulation with *E. coli* [179]. Bister et al. detected MAIT cells in the endometrium of healthy pre- and postmenopausal women, and in decidua from healthy women in first-trimester pregnancies [180]. They showed that endometrial MAIT cells had an activated tissue-resident phenotype by higher expression of CD69, CD103, CD38, HLA-DR, and PD-1 than peripheral blood MAIT cells. Between pairs of monozygotic twins, frequencies of peripheral blood MAIT cells were similar, while frequencies of endometrial MAIT cells varied, suggesting that the migration of MAIT cells to mucosal tissues is influenced by environmental factors. Profiling of endometrial tissue before and after uterus transplantation showed that endometrial MAIT cells were transiently tissue resident, being replaced by MAIT cells that expressed recipient HLA molecules [180]. While this study showed that decidual MAIT cells from healthy women responded to ex vivo stimulation with *N. gonorrhoeae* by expressing CD107a and secreting granzyme B, IFNγ, and TNFα [180], the role of MAIT cells in the in vivo context of STIs of the female genital tract has yet to be elucidated.

## 6. Implications for Vaccine Design

Repeated infections are common for chlamydia, gonorrhea, and syphilis [181,182,183,184], which is evidence for weak acquired natural immunity in humans. This is a major challenge for vaccine development, requiring careful consideration of vaccine platforms, immunogenic antigens, and adjuvants to elicit protective immunity not elicited by the pathogens themselves.

Although acquired immunity from natural infections is weak, there is some evidence for it in the cases of chlamydia and syphilis. For example, prevalence of *C. trachomatis* infection was inversely correlated with increasing age, lower chlamydial burdens were reported in repeat infections, and resistance to reinfection was increased in women who cleared a *C. trachomatis* infection without antibiotic treatment [85,185,186]. We also found that women who had continued exposure after an initial *C. trachomatis* infection but did not experience reinfection over one year had distinct immune profiles, including increased frequencies of CD4 TCM Th1 and Th17 in peripheral blood compared to women who experienced repeat infections [8]. People who became reinfected with syphilis were more likely to have asymptomatic infections and had reduced levels of IL-10 [187,188]. There is no documented evidence of acquired immunity to gonorrhea infections.

The fact that natural infections do not elicit protective immunity in humans creates a challenge for implementing effective vaccines. In fact, natural infections with chlamydia and gonorrhea demonstrate a level of immune evasion by skewing responses toward non-protective phenotypes. Chlamydia infection, for example, drives strong Th2 immunity and antibody responses in humans that are not correlated with protection [6,8,189], whereas Th1 responses are strongly associated with protection in mice and humans but are less frequent in infected individuals [8,88,107]. Similarly, gonorrhea infection drives Th17 responses and depresses Th1/Th2 responses, increasing influx of neutrophils that gonorrhea has mechanisms to resist [23,24]. Traditional vaccines elicit neutralizing antibody responses, which has proven highly effective for some pathogens including HPV (Figure 1). However, antibodies are not associated with protection against chlamydia, and gonorrhea has known mechanisms of antibody evasion, so vaccines that elicit strong T cell-mediated immunity are important for effective protection against bacterial STIs.

While vaccines against chlamydia, gonorrhea, and syphilis are not yet available, there is significant progress being made toward that goal.

### 6.1. Chlamydia Vaccines

Animal models have been essential for chlamydial vaccine research, offering insights into the infection process, immune response, and potential vaccine efficacy before human trials [10,11]. The most used models include mice, guinea pigs, non-human primates, and koalas. Mice, and the murine pathogen *Chlamydia muridarum*, are the most widely used model system for in vivo studies of pathogenesis, immunity, and preclinical evaluation of vaccine efficacy thanks to their relatively low cost and wide range of genetic backgrounds and immunological reagents. Guinea pigs and minipigs have genital tract anatomy more like humans than mice, and also serve as important models to evaluate candidate vaccine efficacy. Non-human primates, such as Cynomolgus and Rhesus macaques, closely mimic human genetics and reproductive physiology, making them ideal for studying transmission, immune response, and long-term protection against human genital infections. Koalas, natural hosts for *Chlamydia pecorum*, provide unique insights into vaccine responses in a naturally susceptible population. Together, these models have contributed to a comprehensive understanding of chlamydial infection and vaccine development.

The first large placebo-controlled human *C. trachomatis* vaccine trials were completed in the 1960s [190,191,192,193,194,195]. They evaluated protection against ocular trachoma with intramuscularly delivered whole-cell vaccines and found short-lived and, in some cases, strain-specific protection. Since then, there has been significant progress in the field toward vaccine development [196].

One chlamydia vaccine is currently being tested in clinical trials. The first Phase 1 clinical trial (NCT02787109) in healthy women evaluated immunization with CTH522, a recombinant version of the major outer membrane protein (MOMP) from *C. trachomatis*, formulated with either the liposome-based cationic adjuvant formulation CAF01 or aluminum hydroxide. Three doses of vaccine were delivered intramuscularly (i.m.) followed by two intranasal (i.n.) doses. The study found that both vaccines were safe and immunogenic, and that the CAF01 formulation elicited higher IgG titers, enhanced mucosal antibody responses, and more consistent cell-mediated immune responses than the aluminum hydroxide formulation [12]. Another recently completed Phase 1 trial (NCT03926728) in healthy men and women evaluated a vaccine consisting of three i.m. doses of CTH522-CAF01 or CTH522-CAF09b followed by intradermal or topical ocular administration of CTH522. The study found that both formulations were safe and immunogenic, and that ocular IgA was induced by a topical ocular booster [13,14]. Future clinical trials will evaluate the protective efficacy of CTH522-based vaccines against both ocular and urogenital *C. trachomatis* infections.

Many other candidate vaccines are in the preclinical development phase in animal models and have been recently reviewed [15,16]. Primary infection of mice with virulent or attenuated *C. muridarum* provides sterilizing but short-lived immunity against subsequent challenge [10,197,198]. MOMP is the most popular protein target for candidate vaccines, as it makes up approximately 60% of the protein mass of the chlamydial outer membrane [199]. However, monomeric MOMP is challenging to purify from cells. Chlamydial protease-like activation factor (CPAF) and polymorphic outer membrane proteins (Pmp) are other immunogenic proteins that have demonstrated protection against chlamydial challenge in mice [200,201,202,203,204,205,206]. It is also increasingly evident that adjuvants that support Th1 immunity and mucosal routes of immunization may be required for optimal protection [17,108,167,168,207,208].

### 6.2. Gonorrhea Vaccines

Preclinical testing of *N. gonorrhoeae* vaccines has been limited by the difficulty of adapting studies of the human-restricted pathogen to animal models [26,27]. However, treating female mice with estradiol to extend estrus and antibiotics to suppress the overgrowth of commensal flora allows colonization of the genital tract with *N. gonorrhoeae* [28], and such models have improved the ability to study *N. gonorrhoeae* biology and immunity in vivo. Limitations to these models still exist since *N. gonorrhoeae* uses human-specific receptors for adherence and invasion pathways, some of which can be addressed by using humanized transgenic mice [209,210]. The *N. gonorrhoeae-*controlled human infection model (Ng CHIM) is also a valuable model for studying gonorrheal pathogenesis and immunity, but it is only used for male urethral infection due to risk of pathogen ascension in the female genital tract [29]. The mouse and Ng CHIM models are both useful in studies of correlates of immunity and vaccine testing.

There are multiple gonorrheal vaccines in clinical trials. Of note, the US Food and Drug Administration (FDA) granted fast-track designation to GSK’s generalized modules for membrane antigen (GMMA)-based Neisseria Gonorrhoeae GMMA (NgG) vaccine in 2023. GMMA vaccines use genetically modified Gram-negative bacterial pathogens to overproduce outer membrane vesicles (OMV), which are usually produced at relatively low amounts, increasing yield and reducing LPS endotoxicity for upscaled production [30]. The currently ongoing clinical trial (NCT05630859) is a Phase 1/2 trial to evaluate safety and efficacy. Phase 1 was completed with positive safety results and Phase 2 is ongoing, so the immunogenicity and efficacy of the vaccine is yet to be determined. Most other completed and ongoing clinical trials are focused on evaluating the cross-protective efficacy of the currently licensed meningococcal group B vaccine 4CMenB (Bexsero, GSK) against *N. meningitidis* and have been reviewed recently [31,211]. 4CMenB is composed of (1) fHbp variant 1.1 (subfamily B) fused to the genome-derived *Neisseria* antigen (GNA) 2091; (2) *Neisseria* adhesin A (NadA); (3) Neisserial heparin binding antigen (NHBA) peptide 2 fused to GNA1030; and (4) the MeNZB vaccine, composed of *N. meningitidis* OMVs from a strain isolated in New Zealand containing porin A (PorA) P1.4. Although not all these antigens are homologous to *N. gonorrhoeae*, NHBA is 68.8% identical, and many of the New Zealand-strain OMV proteins share >90% homology [212], providing opportunity for cross-protection. Serum from Bexsero-immunized people contained antibodies that recognized gonorrheal antigens [213]. Real-world evidence of 4CMenB efficacy against gonorrheal infection includes a study in Canada that found estimated risk reduction of 59% [214], a study in the US that reported vaccine efficacy of 40% [215], and a study in South Australia that found vaccine efficacy of 33% [216]. Recently completed clinical trials (NCT04722003, NCT04094883) evaluated the systemic and local immune responses against *N. gonorrhoeae* following 4CMenB vaccination, but published results are not yet available. These studies will be important to identify correlates of immunity and to improve vaccines targeted against *N. gonorrhoeae* infections. Studies in mice showed that 4CMenB vaccination accelerated clearance and reduced *N. gonorrhoeae* bacterial burden in the female genital tract and elicited vaginal IgG and IgA. However, T cell responses against *N. gonorrhoeae* elicited by 4CMenB vaccination have yet to be evaluated in mice or humans and are needed considering protection against *N. gonorrhoeae* in mice is associated with the Th1 immune response [25]. Indeed, intravaginal vaccination of mice with an experimental *N. gonorrhoeae* OMV vaccine and coadministration of IL-12 generated IFNγ-producing CD4 T cells and accelerated bacterial clearance following *N. gonorrhoeae* challenge [33]. Investigation of potential recombinant antigens for *N. gonorrhoeae* subunit vaccines is also ongoing [32].

### 6.3. Syphilis Vaccines

Syphilis vaccine research is also limited by the lack of in vitro and animal model systems to study *T. pallidum*. In vitro culture of the organism was first reported in 2018 [39]. Studies of *T. pallidum* pathogenesis and immunity are primarily conducted in rabbits [38], but immunological reagents are severely lacking for this model system. Non-human primates and hamsters have also been used to model *T. pallidum* infection [217,218], but these studies are limited. A C57BL/6 model was recently discovered and has potential to be a useful tool for future immunological research, but infected mice lack clinical manifestations [40].

Heat- or penicillin-inactivated *T. pallidum* was shown to provide partial protection in rabbits [219]. However, the fragility of the *T. pallidum* outer membrane and the lack, until recently, of in vitro models for production of large quantities of organisms for use in vaccines has limited their study. Experimental DNA vaccines have demonstrated attenuated lesions and decreased bacterial burden in rabbit models [220,221,222], and experimental recombinant protein vaccines have demonstrated some success as well (recently reviewed in [223]). So far, none of these vaccines have moved forward to human trials, but the recent advances in in vitro culture and animal models will be important tools for future immunological evaluation of candidate vaccines.

## 7. Conclusions

Prevalence of bacterial sexually transmitted infections (STIs) chlamydia, gonorrhea, and syphilis is inversely proportional to the occurrence of symptoms. These infections not only impact sexually active adolescents and adults but also pose serious risks to newborns through mother to child transmission. Current public health interventions include use of barrier methods, doxycycline post-exposure prophylaxis for men who have sex with men (MSM), and increased diagnostic surveillance and reporting [45], but effective vaccines are not available for disease prevention. A significant challenge in understanding immunity to these infections is that natural infection does not confer strong protective immunity, as these pathogens have developed mechanisms to evade both innate and adaptive host immune responses. This review focuses on adaptive immunity, but innate immune evasion by these pathogens has been reviewed elsewhere [32,224,225,226]. These immune evasion mechanisms underscore the importance of generating protective T cell responses with effective vaccines. Recent advances in understanding the quality of cellular responses needed, such as CD4 Th1 responses for *C. trachomatis* and *N. gonorrhoeae*, and the availability of Th1-inducing adjuvants, offer hope for the development of effective vaccines. Additionally, the establishment of models for testing syphilis vaccines represents a critical step forward. These aspects together, as summarized in Table 1, demonstrate that continued research into the immunological mechanisms is essential for continued vaccine development.

## Figures and Tables

**Table 1:** A summary of immune responses to and vaccine progress against bacterial STIs.

Disease [*Pathogen*]	Symptoms of Female Genital Tract Infection	Immune Evasion Mechanisms	Protective Immune Responses	Experimental Models	Vaccine Development
Chlamydia *[Chlamydia trachomatis]*	Lower genital tract discharge, dysuria, dyspareunia, urethritis, menstrual irregularities, PID; long term complications include chronic pelvic pain, infertility, ectopic pregnancy, adverse pregnancy outcomes Often asymptomatic Mother to child transmission during birth can cause neonatal conjunctivitis and pneumonia	Evades recognition in intracellular niche [4] Can persist within inclusion in a low metabolic state [4] Multiple molecular mechanisms of epithelial cell invasion [4] Inhibition of neutrophil functions [5] Responses to natural infection are skewed toward Th2 in humans, supporting antibody production which is not associated with long-term protective immunity [6,7,8]	IFNγ-mediated tryptophan depletion in humans restricts chlamydial growth and replication [9] CD4^+^ Th1 and Th17 responses associated with reduced reinfection in humans [6,8]	Mice, guinea pigs, non-human primates, koalas [10,11]	Recombinant MOMP vaccine adjuvanted with CAF01 in Phase 1 human clinical trials (NCT02787109, NCT03926728) [12,13,14] Several preclinical vaccine candidates using MOMP, CPAF, PmpG recombinant proteins with an emphasis on Th1-inducing adjuvants [15,16,17]
Gonorrhea *[Neisseria* *gonorrhoeae]*	Lower genital tract discharge, dysuria, dyspareunia, urethritis, menstrual irregularities, PID; long term complications include chronic pelvic pain, infertility, ectopic pregnancy, adverse pregnancy outcomes Often asymptomatic Disseminated infections can lead to sepsis, skin rash, arthritis, endocarditis, meningitis Mother to child transmission during birth can cause ocular or disseminated infection	Antigenic and phase variation [18] Induces IL-10 and regulatory T cells that suppresses antibody responses [19] Inhibits complement deposition and blocks activation of complement [20,21] Epitope masking by IgA1 protease [22] Natural infection in mice drives Th17 and neutrophil responses, which *N. gonorrhoeae* resists, while suppressing protective Th1 responses [23,24]	CD4^+^ Th1 responses associated with protection in mice [25]	Antibiotic-treated mice, humanized mice [26,27,28] Controlled human infection model (CHIM) [29]	Phase 2 and Phase 4 human clinical trials evaluating efficacy of currently licensed meningococcal vaccine 4CMenB (Bexsero, GSK) against *N. gonnorrhoea* infections (NCT04722003, NCT04094883) [30,31] OMV-based vaccine in Phase1/2 human clinical trials (NCT05630859) Pre-clinical studies evaluating OMV-based vaccines in mice and determining candidate antigens for recombinant protein vaccines [32,33]
Syphilis *[Treponema* *pallidum]*	Primary stage: chancre at infection site Secondary stage: skin lesions, fever, fatigue, rash, muscle aches, weight loss, headaches, hair loss and swollen lymph nodes Tertiary stage: neurological and cardiovascular complications, seizures, deafness, and vision problems including blindness Congenital syphilis: severe outcomes in newborns including prematurity, low birth weight, pneumonia, rash, neurologic disease, and bone abnormalities	Ability to remain dormant for long periods Antigenic variation [34]	CD4^+^ Th1 IFNγ-mediated recruitment of macrophages [35] Macrophage mediated opsonophagocytosis and killing [36,37]	Rabbits are most widely used [38] Recent development of mouse and in vitro models [39,40]	Pre-clinical studies identifying potential target antigens for recombinant protein vaccines [35,41]
